# Use of pre-operative 3D planning software for revision shoulder arthroplasty: clinical experience data from a survey in a real-world setting

**DOI:** 10.1016/j.xrrt.2024.05.010

**Published:** 2024-06-16

**Authors:** Matthias Regling, Brendan M. Patterson

**Affiliations:** a Stryker Trauma GmbH, Schönkirchen, Germany; b Department of Orthopedics and Rehabilitation, University of Iowa, Iowa City, IA, USA

**Keywords:** Shoulder arthroplasty, Total shoulder arthroplasty, Reverse shoulder arthroplasty, Revision shoulder arthroplasty, Preoperative planning, Preoperative planning software, 3D, Computed tomography

## Abstract

**Background:**

Preoperative 3D planning is routinely used in primary shoulder arthroplasty, while specific challenges in the revision setting make such approaches more cumbersome and less accessible. Recently, an established preoperative planning software (Blueprint; Stryker, Tornier SAS, Montbonnot-Saint-Martin, France) was expanded to offer a capability for planning of revision and complex primary shoulder arthroplasty cases. The aim of this study was to survey experienced surgeons on their perception of the new software feature for preoperative 3D planning in the setting of revision shoulder arthroplasty.

**Methods:**

An observational survey was conducted from January 2022 to October 2022 among orthopedic surgeons performing revision shoulder arthroplasty cases. The survey was part of the Early Product Surveillance program, with the primary goal of obtaining observational data from surgical experience in a real-world setting. A two-staged survey process was applied with separate questionnaires to seek voluntary feedback on the preoperative planning phase as well as on the intraoperative evaluation of the software planning features in revision shoulder arthroplasty.

**Results:**

Twenty-five fellowship-trained orthopedic surgeons from the USA and Canada participated in the survey and reported their feedback on 34 revision shoulder arthroplasty cases that were preoperatively planned with the use of Blueprint revision planning software. The surgeons were largely in favor of the revision software planning features and confirmed perceived benefits of its use in the preoperative planning stage of revision shoulder arthroplasty cases. Reported benefits in the preoperative planning phase included increased efficiency and improved ease of creating an appropriate surgical plan as well as increased confidence to execute revision shoulder arthroplasty cases. Surgeons also noted improvements in translation of preoperative planning to intraoperative execution of revision cases, including more appropriate implant selection and improved accuracy of implant placement.

**Conclusion:**

The feedback from fellowship-trained shoulder arthroplasty surgeons on the use of the new software feature for preoperative 3D planning of revision shoulder arthroplasty is largely favorable. Further research should be conducted to investigate whether these surgeon-perceived benefits can lead to improved clinical outcomes for patients.

In recent years, the annual procedural volume of shoulder arthroplasty in the United States of America (USA) has increased.^7^^,^^15^^,^^25^ Since the USA, like many other countries, is facing an aging population^3^ and the incidence of shoulder arthroplasty peaks in patients aged 75-84^8^, this trend is likely to continue. With an increasing number of primary shoulder arthroplasties, it is apparent that the number of revision shoulder arthroplasties will also increase substantially in the coming years. Meta-analyses including older studies found high revision rates of 8% for anatomic total shoulder arthroplasty (aTSA)^11^ and 10% for reverse total shoulder arthroplasty (rTSA),^26^ although these high rates appear to have improved in more recent studies.^10^ Revision surgery also shows a tremendous increase in costs. The national cumulative cost of revision surgery increased from $26 million to $206 million from 2002 until 2017 in the USA.^9^

Adding to the increased burden of cases and the increasing costs, it is well known that the outcomes of primary surgery are often more predictable and successful as compared to revision surgery.^26^ In a systematic review of the literature published in the USA and Europe, an overall complication rate of 17% was found for revision shoulder arthroplasty, with a total of 465 described complications. Of those 465 described complications, 171 (37%) led to reoperations and a total of 162 (35%) resulted in a secondary revision procedure.^16^ Another study found a rerevision rate of 13% after 2 years, which increased to 35% at 5 years.^19^

Preoperative planning software solutions have emerged as a useful tool in the decision-making process for surgeons performing primary shoulder arthroplasty and are becoming increasingly popular – at least 33% of all aTSA patients receive some type of 3D imaging preoperatively, and lower revision rates have been reported for patients who undergo a computed tomography (CT) scan.^5^ For revision surgery, however, there is an increased difficulty in processing images due to image artifacts from in-situ implants and difficulty to discern the different areas of cement and polyethylene components. Additionally, the estimation of bone loss and bone quality can also be difficult to determine from the preoperative CT scan in the revision setting.

In view of the aforementioned trends and current inadequacies, it is of great importance that new technologies are made available to support surgeons in their decision-making in revision arthroplasty. Stryker has recently expanded their Blueprint software to include a preoperative planning capability for revision and complex primary cases. The related process includes a manual segmentation of the CT scan (performed by a Blueprint software technician), after which the surgeon receives a 3D rendering to virtually plan a case with implantation of virtual components (Fig. 1).^23^ This study discusses the first user experience of 34 revision shoulder arthroplasty cases that were planned utilizing the new Blueprint Revision and Complex Cases planning software feature (Stryker, Tornier SAS, Montbonnot-Saint-Martin, France).

**Figure 1:**
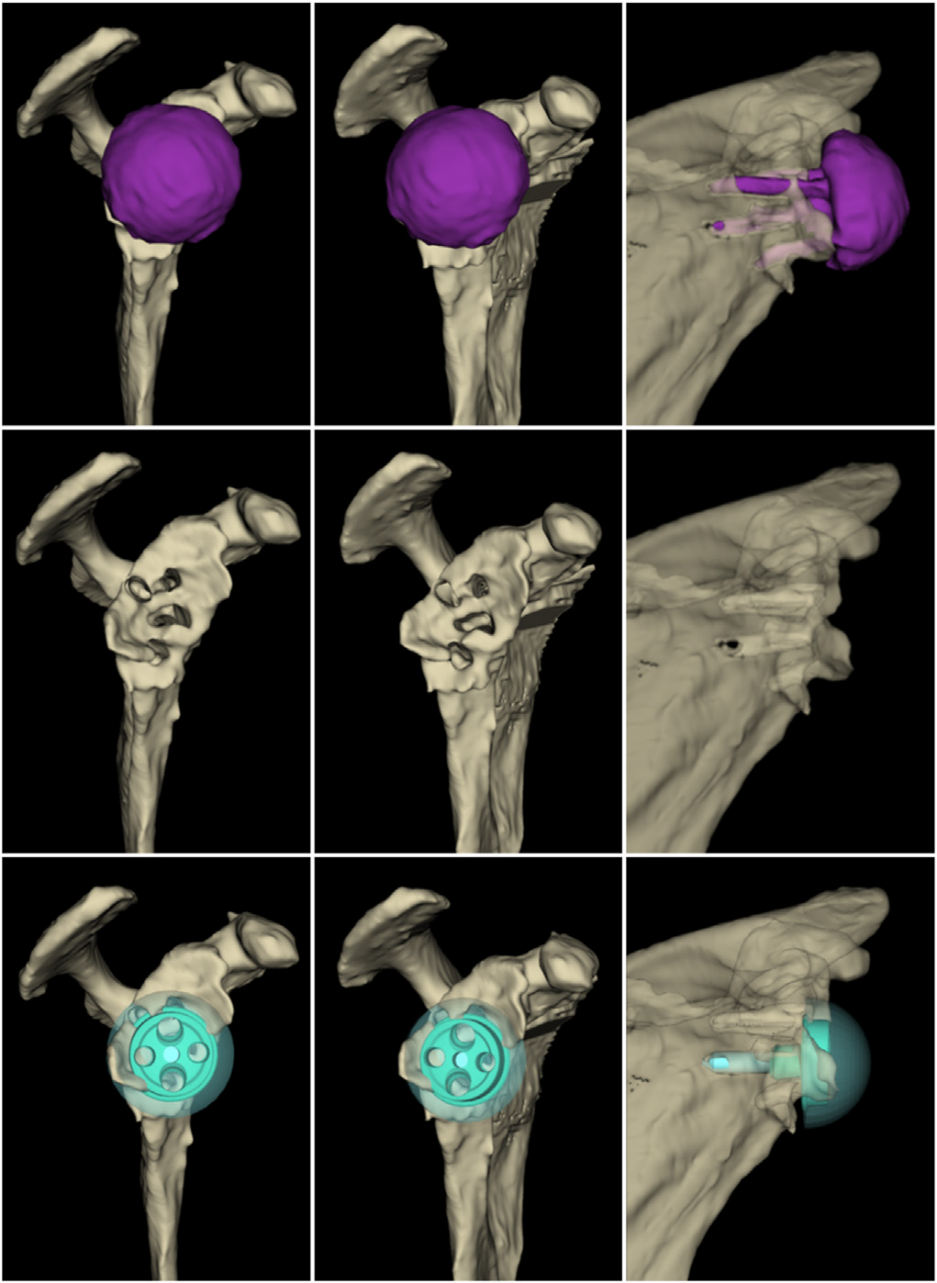
Screenshots taken from the Blueprint revision and complex cases planning software feature, showing different views of 3D renderings of the glenoid side for the preoperative planning of a revision shoulder arthroplasty case (rTSA to rTSA). **Top row:** segmented in-situ implants; **Middle row:** virtually removed implants to assess cavities in bony structures left from prior implants; **Bottom row:** virtually placed new implants. *rTSA*, reverse total shoulder arthroplasty.

## Materials and methods

From January 2022 to October 2022, a survey was conducted among U.S. and Canadian surgeons after receiving standard market approval for the Blueprint Revision and Complex Case planning software feature from respective authorities (U.S. Food and Drug Administration, Health Canada). The survey was set up as an observational research study to collect voluntary surgeon feedback on technical and procedure-related aspects of the new software feature for revision shoulder arthroplasty cases.

A two-staged survey process was applied with separate questionnaires, which covered an evaluation of the preoperative planning in the Blueprint software (part 1) as well as case details, an intraoperative evaluation of how the new feature supported the execution of the surgical procedure and a general feedback section (part 2). Specific questions in the part 1 survey included basic demographic questions of the participating surgeons, such as questions pertaining to annual procedural volumes and the overall use of preoperative planning software in shoulder arthroplasty cases. In addition, respondents were asked to evaluate the software feature on criteria such as the time spent planning the case, satisfaction with the lead time and quality of the manual segmentation, as well as overall satisfaction and evaluation of ease of use. Furthermore, surgeons were asked how they planned revision shoulder arthroplasty cases prior to having access to this software and how this compared to the newly available feature. Part 2 of the survey focused on the intraoperative evaluation, and respondents were asked to evaluate how the Blueprint plan supported them during the surgical procedure. Specifically, feedback was obtained on criteria such as comparing the plan to the patient’s anatomy and in-situ implants, intraoperative implant type and/or size changes (as compared to the plan), as well as a comparison to traditional surgery (without use of preoperative planning software) along a variety of different aspects. Respondents were also given the option to comment in open field sections as they saw fit, including on their subjective opinion regarding main benefits and drawbacks of the new software feature.

The web-based questionnaire was deployed using the Qualtrics survey platform, version November 2021 (Qualtrics, Provo, UT, USA). Hard copy forms were offered and distributed via email. Coordination of the return of completed responses was facilitated by medical device sales representatives, who were trained on the new software feature as well as the process of response return.

Open-answer fields related to subjective opinions on the main benefits and drawbacks of the system were thematically coded. Qualitative data were analyzed as relative frequencies; quantitative data were examined for central tendency and dispersion. Unanswered survey items were treated as missing data. In addition, the imaging files that were uploaded into the software were systematically analyzed with respect to the details of the planned revision procedures (i.e., original vs. planned revision implant construct) as well as the extent and severity of the humeral and glenoid bone loss according to established classification systems (Proximal Humeral Arthroplasty Revision Osseous inSufficiency [PHAROS] classification system,^6^ Antuna classification of glenoid bone loss^1^) to further characterize the revision cases undergoing preoperative 3D planning in the new software feature.

## Results

### Demographics

A total of 25 individual surgeons, 22 from the USA and 3 from Canada, participated in the survey regarding their experience with the new software feature when used in the revision shoulder arthroplasty setting. All surgeons completed fellowship programs with training in shoulder arthroplasty and had previously utilized the Blueprint preoperative planning software for primary shoulder arthroplasty cases (Table I).

**Table I:** Surgeons’ training and experience.

Fellowship specialty	n (%)
Completion of one fellowship program	
Shoulder and elbow/upper extremity surgery/reconstruction	14 (56)
Orthopedic sports medicine	4 (16)
Combined sports medicine and shoulder surgery/reconstruction	4 (16)
Completion of two fellowship programs	
Shoulder and elbow surgery/reconstruction AND sports medicine	3 (12)

Time in practice	median (range) [y]

U.S. surgeons: time since completion year of fellowship program	10 (3-22)
Canadian surgeons: time since FRCSC designation	18 (16-22)

Annual volume of shoulder procedures	median (range) [cases]

aTSA	30 (10-120)
rTSA	105 (25-280)
Revision cases	15 (3-60)

Regular use of Blueprint in primary shoulder arthroplasty (% of cases planned with Blueprint)	median (range)

aTSA	75% (0-100%)
rTSA	75% (5-100%)

*FRCSC*, fellows of the royal college of surgeons of Canada; *rTSA*, reverse total shoulder arthroplasty; *aTSA*, anatomic total shoulder arthroplasty.

Percentages may not equal 100% because of rounding.

The imaging files that were uploaded into the software for the purposes of preoperative planning were systematically analyzed with respect to the details of the planned revision procedures (i.e., original vs. planned revision implant construct, Table II), as well as the extent and severity of the humeral and glenoid bone loss and structural gaps left by previous implants (Table III). According to the preoperative plans completed by the surgeons in the software, the planned revision procedures entailed the removal of a humeral implant component (incl. osteosynthesis devices, where applicable) in most cases (34/36 cases, missing data in the remaining 2 cases).

**Table II:** Overview of original vs. planned revision implant constructs.

	n (%)
HA to rTSA	7 (19)
Of these, stemless HA to rTSA	1 (3)
aTSA to rTSA	13 (36)
Of these, stemless aTSA to rTSA	2 (6)
rTSA to rTSA	10 (28)
Intramedullary nail to rTSA	1 (3)
Plate to rTSA	1 (3)
Missing data	4 (11)

*HA*, Hemiarthroplasty; *rTSA*, reverse total shoulder arthroplasty.

Percentages may not equal 100% because of rounding.

**Table III:** Classification of humeral and glenoid bone loss.

Humeral bone loss	n (%)
PHAROS Classification System^6^^,^∗	
Type 1	11 (31)
Of these, subtype 1C	7 (19)
Of these, subtype 1G	1 (3)
Type 2	3 (8)
Of these, subtype 2A	3 (8)
Type 3	0 (0)
No humeral bone loss	20 (56)
Missing data	2 (6)

Glenoid bone loss	n (%)		
Antuna Classification System^1^	Mild	Moderate	Severe
Central	1 (3)	14 (39)	3 (8)
Peripheral (anterior posterior)	0 (0)	0 (0)	0 (0)
Combined	0 (0)	6 (17)	7 (19)
No glenoid bone loss		3 (8)	
Missing data		2 (6)	

*PHAROS*, Proximal Humeral Arthroplasty Revision Osseous inSufficiency.

Percentages may not equal 100% because of rounding.

∗ Type 1 = epiphyseal bone loss (Type 1C with calcar compromise; Type 1G with greater tuberosity compromise); Type 2 = metadiaphyseal bone loss above the deltoid attachment (Type 2A with cortical thinning of the metadiaphysis >50% of the expected cortical thickness based on the noninstrumented portion of the humerus with associated epiphyseal loss or cortical thinning); Type 3 = diaphyseal bone loss extending below the deltoid attachment.

### Preoperative planning – part 1 survey results

A total of 36 responses were collected in the preoperative planning phase, in which surgeons utilized the new Blueprint functionality of revision planning. The process of submitting a new case was rated positively regarding intuitiveness in 97% of cases (missing data in the remaining case). Furthermore, surgeons gave positive feedback on the lead time of the manual segmentation in 75% of cases, whereas dissatisfaction was raised in 8% of cases, with surgeons commenting overall that they would have preferred shorter lead times (neutral ratings in the remaining cases). For comparison, the average lead time for the overall period during which the survey was conducted was 3.3 days, with monthly average lead times ranging from 2.1 to 5.7 days. When asked about the quality of the manual segmentation and the visibility of anatomical structures, surgeons indicated to be either ‘satisfied’ or ‘completely satisfied’ in 95% and 97% of cases, respectively (neutral ratings in the remaining cases). Surgeons indicated that there were no artifacts obstructing the view or impairing the surgeon’s ability to plan the case in 94% of cases. In most cases, surgeons were able to complete the preoperative planning with Blueprint in 20 minutes or less (Table IV), and all surgeons confirmed their satisfaction with the time spent and efficiency of case planning with the new software feature.

**Table IV:** Time spent for preoperative case planning.

	n (%)
Less than 10 min	11 (31)
10-20 min	22 (61)
20-30 min	2 (6)
More than 30 min	1 (3)

Percentages may not equal 100% because of rounding.

**Figure 2:**
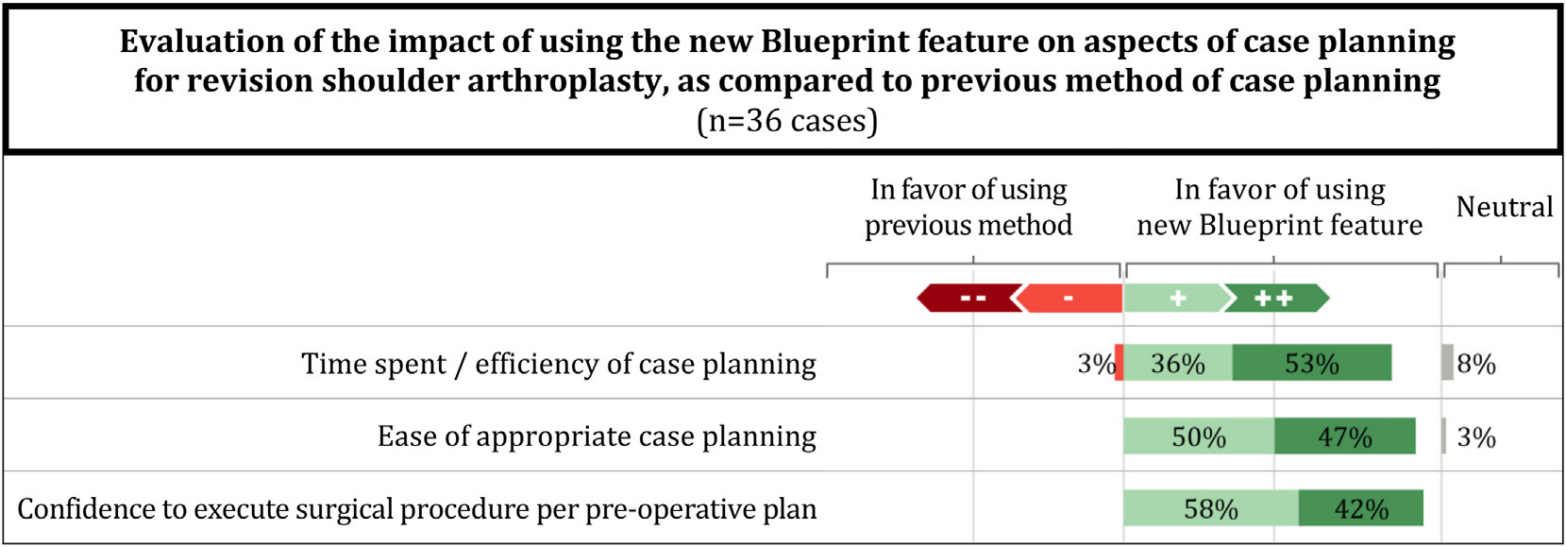
Comparative evaluation of the new software feature vs. the surgeons’ previous method of planning for revision shoulder arthroplasty.

Respondents were further asked to compare the new software feature against their previous approach to planning for revision shoulder arthroplasty, with the results being largely in favor of the new Blueprint feature (Fig. 2).

**Figure 3:**
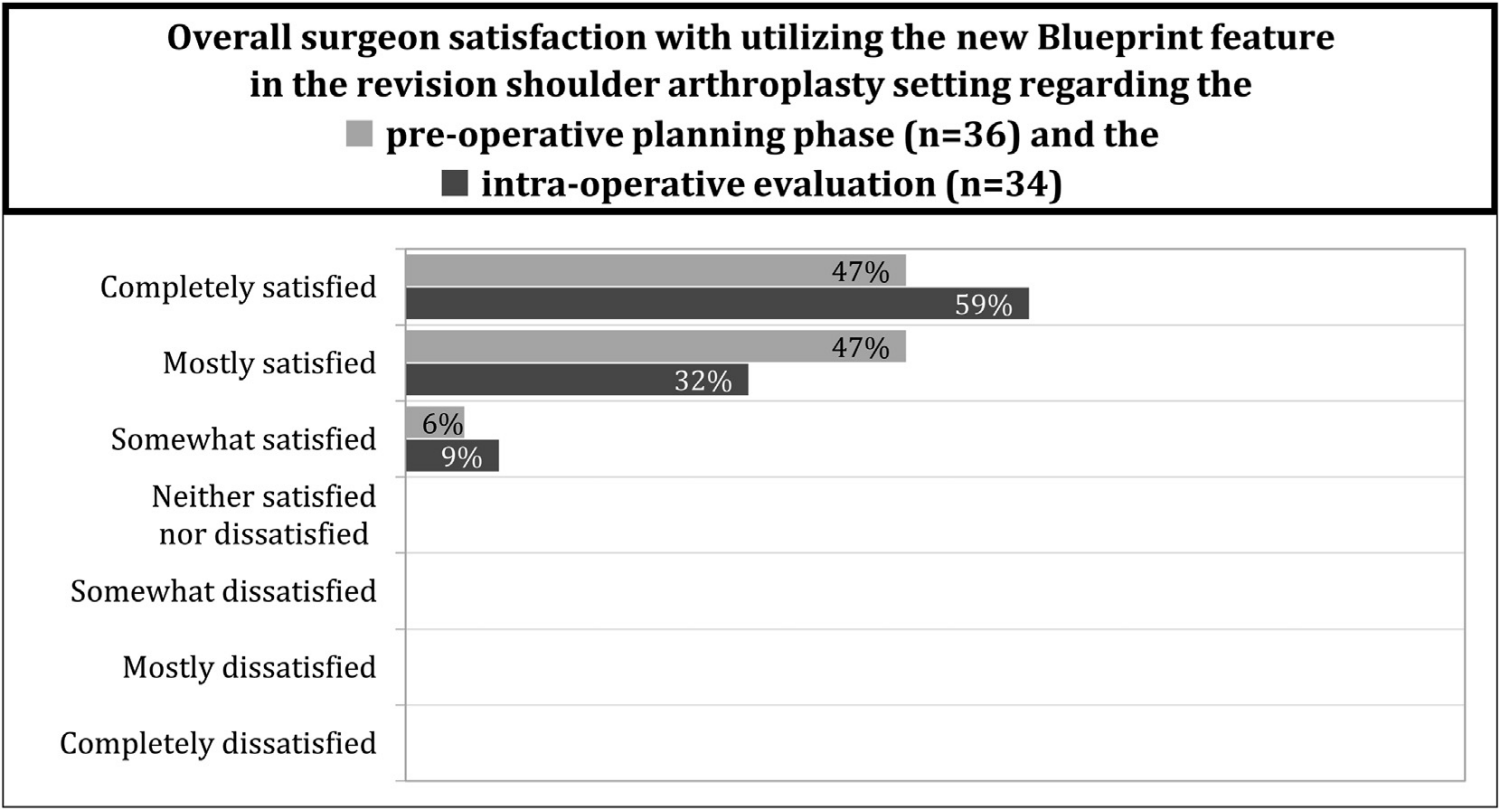
Overall surgeon satisfaction with utilizing the new Blueprint feature in the revision shoulder arthroplasty setting regarding the preoperative planning phase (n = 36) and the intraoperative evaluation (n = 34).

Overall surgeon satisfaction with the preoperative planning in Blueprint was high, with surgeons responding on the positive side of the satisfaction scale in all cases (Fig. 3). Likewise, the surgeons confirmed the ease of use of case planning in all reported cases.

### Intraoperative utility – part 2 survey results

Respondents who completed part 1 of the survey were asked to complete part 2 of the survey after having performed the revision shoulder arthroplasty. A total of 34 responses were captured from 23 individual surgeons. The median duration of the procedure was 1 hour and 30 minutes (range: 1 hour-3 hours and 30 minutes).

The comprehensiveness of the Blueprint planning report was rated favorably in the majority of cases (94%; missing data in the remaining cases). In addition, respondents confirmed that the Blueprint plan gave an accurate reflection of the patient’s anatomy and in-situ implants and that it helped them obtain additional information as compared to 2D images only in most of the cases (91% and 85%, respectively).

As part of the Blueprint plan created preoperatively, the surgeons selected the implant type and size that was deemed best suited for the procedure. When deciding for the final implant construct in the operating room (OR), the surgeons adhered to this plan for both the implant type and size in most cases (Table V). Reported reasons for opting for another implant type included, among others, periprosthetic fractures (e.g., upon removal of in-situ implant constructs), the intraoperative decision to leave a convertible stem in as well as variations in glenoid bone stock or glenoid vault characteristics. The reasons for implant size changes were, e.g., related to upsizing or downsizing the implant diameter for an optimized press-fit or soft-tissue tensioning.

**Table V:** Adherence to preoperative plan for selected implants.

	Implant type, n (%)	Implant size, n (%)
Adherence to preoperative plan	25 (74)	24 (71)
Selection of another implant	9 (26)	10 (29)
Total		
Humeral implant	5 (15)	7 (21)
Glenoid implant	3 (9)	1 (3)
Both humeral and glenoid implants	1 (3)	2 (6)

Percentages may not equal 100% because of rounding.

**Figure 4:**
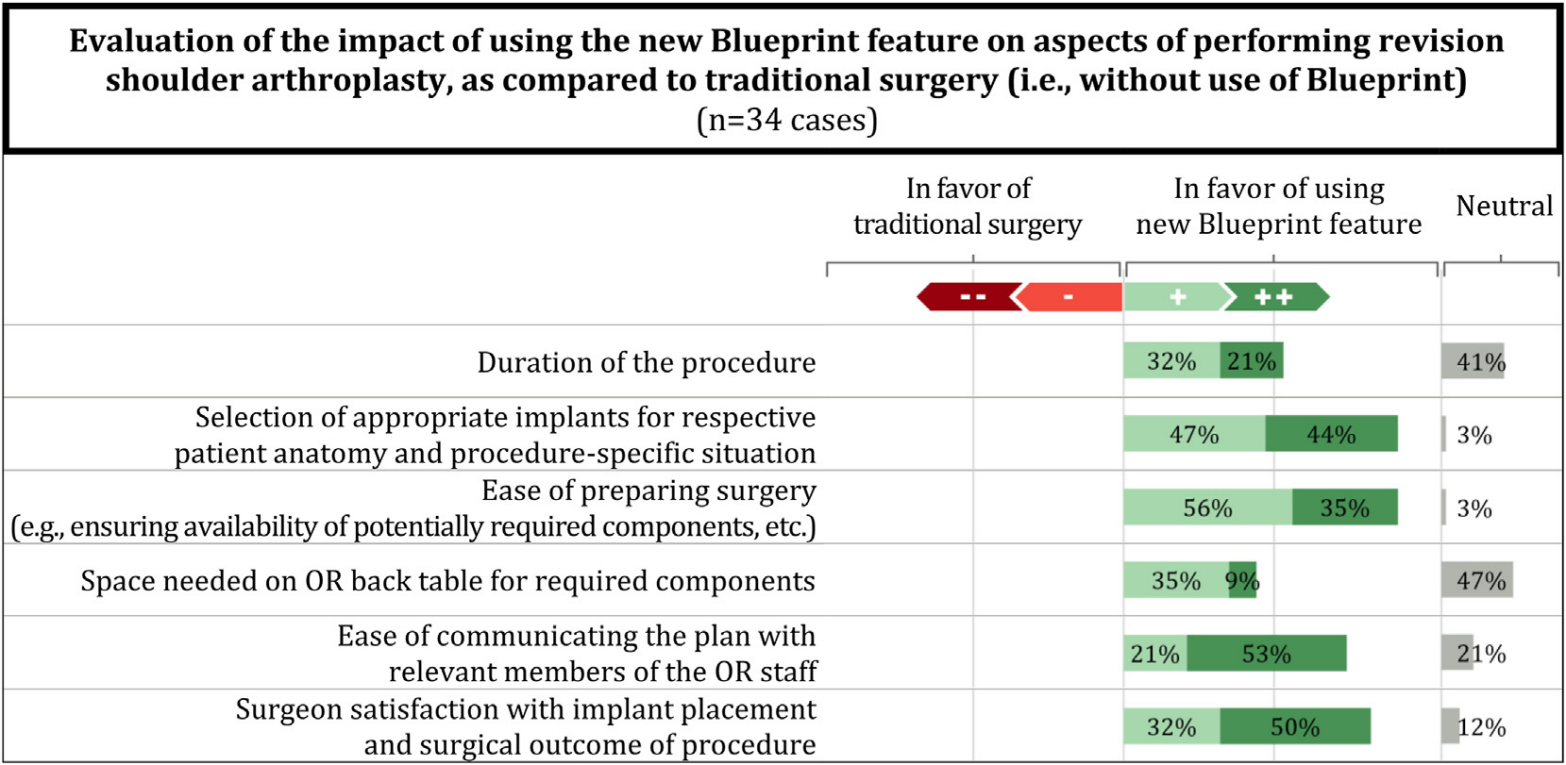
Comparative evaluation of the new software feature vs. traditional surgery (i.e., without use of Blueprint; values not displayed (i.e., where sum of values is less than 100%) are related to missing data or ‘no-answer’ responses).

In addition, the participating surgeons were asked to evaluate the impact the use of the new Blueprint feature in the preoperative planning phase has on a variety of different aspects of shoulder arthroplasty when compared to traditional surgery (i.e., without the use of Blueprint). The surgeons rated these predominantly in favor of the new software feature (Fig. 4). Efficiency gains regarding the duration of the procedure and the space needed on the OR back table for required components were further quantified by the respondents. According to the surgeons and across all cases (including those with neutral ratings, where time or space savings were set equal to 0), the preoperative plan from Blueprint helped surgeons gain time savings in the OR of 10 minutes (median; range: 0-30 minutes), while space savings were quantified as neutral (median of 0%; range: 0%-50%).

In addition to the favorable comparative evaluation vs. traditional surgery, the feedback from surgeons about future use is underlining the acceptance of the new software feature: in 95% of the cases, surgeons stated they would be ‘likely’ or ‘very likely’ to use Blueprint revision and complex case planning in the future and likewise confirmed they would recommend it to others in 97% of cases (missing data in the remaining cases). Moreover, respondents affirmed in 79% of the cases that they would use the new software feature for every applicable procedure, and in 88% of cases that they consider utilizing Blueprint in training others outside the OR.

Overall qualitative surgeon satisfaction with the new revision software planning feature was positive, with respondents answering on the positive side of the satisfaction scale in all cases (Fig. 3). Similarly, the surgeons rated the overall ease of use and usability aspects favorably in all cases.

Moreover, the comments made by the surgeons on their subjective opinions regarding main benefits and drawbacks of the new software feature yielded mostly positive results, while some drawbacks were also noted (Table VI). Comments on drawbacks were carefully examined and taken into consideration in the context of continuous product improvement.

**Table VI:** Surgeons' perception of main benefits and drawbacks of the new Blueprint feature in the revision setting (multiple answers allowed per respondent).

Main benefits	n [# of mentions]
Improved visualization and greater understanding of anatomical structures (e.g., atypical wear pattern, glenoid deformity, cavities in bony structures resulting from previous implants, points of reference for implant positioning) and in-situ implants	18
Improved implant selection based on virtual trialing of planned case (incl. removal of implants to be revised, evaluation of fit of new implants, etc.)	14
Improved implant placement (e.g., entry point and trajectory of the guide pin, maximizing impingement-free range-of-motion, avoidance of dangerous bone defects)	10
Ease of preparing for surgery (i.e., ensuring that all potentially required devices and implants are available)	2
Ability to communicate the plan with other physicians and the surgical team prior to the procedure	1
Ability to communicate the plan with the patient	1
Increased confidence in the pre-operative plan	1

Drawbacks	n

Lack of support for a patient-specific instrument guide in revision and complex cases	3
Limitations in information available for glenoid measurements (in comparison to normal scope of information available in Blueprint, e.g., in terms of the contact map and the amount of reaming needed)	3
Suboptimal lead time for the manual segmentation	2
Suboptimal planning capabilities for the humeral side (i.e., rotation of planned stem, consideration of height and version)	2
Lack of support for the management of soft tissues	1
Suboptimal accuracy of the manual segmentation	1
Suboptimal default plan	1

## Discussion

Revision shoulder arthroplasty cases are increasing in numbers.^9^^,^^11^^,^^26^ Standard 2D imaging is often not adequate to acquire a comprehensive understanding of the patient’s anatomy and complex bone morphology^21^; hence, 3D preoperative case planning has evolved as a trending topic and is increasingly finding its way into practice.^5^ In revision surgery, however, there are only a few commercially available software solutions to date: some of these are dedicated to custom-made implants that may require a two-stage revision procedure, whereas some well-resourced healthcare institutions have developed their own in-house applications; stand-alone software with a specific focus on preoperative 3D planning of revision procedures is still rarely offered.^23^ Against this background, it is unsurprising that as noted in a recent review article, “literature regarding digital templating in the revision setting is scarce and represents a promising area of future study”.^18^ Our article contributes to the limited body of evidence in reporting clinical experience data on the use of preoperative 3D case planning for revision shoulder arthroplasty. Although we did not look at clinical results of utilizing Blueprint for revision shoulder arthroplasty, our findings give insights into how surgeons perceive the new software feature and how it could support their current practice. Overall, the results of the survey showed that surgeons found the addition to the software to be valuable and most will continue to utilize it in the future. For the interpretation of our results that are based on surgeon preferences, it is relevant to consider the high experience levels of responding surgeons (Table I).

The most noteworthy results of our survey include the surgeons’ feedback on implant selection and placement. Even though respondents indicated in some cases that they decided in the OR to change the implant size and/or type from the preoperative plan, they confirmed that the selection of implants was ‘better’ or ‘much better’ with the use of revision planning software in comparison to traditional surgery in 92% of cases. For primary arthroplasty procedures, previous studies have attested to preoperative planning software solutions a high concordance between plan and final implants, both in aTSA and in rTSA settings.^2^^,^^17^ Likewise, the surgeons responding to our survey stated to be ‘more satisfied’ or ‘much more satisfied’ with the implant placement and the surgical outcome of the procedure due to the use of the new Blueprint feature in 82% of cases. The positioning of implants, especially of the glenoid component, and the optimization of the interplay between implant components have a crucial role and can impact clinical outcomes by avoiding known complications such as scapular notching and subacromial impingement and their repercussions.^4^^,^^20^^,^^22^

As another important finding, all surgeons confirmed in all cases that the preoperative planning in Blueprint helped them to feel ‘more confident’ or ‘much more confident’ to execute the surgical procedure per plan, as compared to their previous method of case planning. A recent survey study among orthopedic surgeons provided evidence that the use of clinical decision support tools increases surgeon confidence when indicating primary cases for either aTSA vs. rTSA treatment.^24^ With the new software feature in Blueprint, a comprehensive case planning functionality is now also available for revision and complex cases, representing an important addition to the armamentarium of clinical decision support tools.

Furthermore, the results of our survey suggest that use of Blueprint for revision cases increases both the ease and the efficiency of appropriate case planning. Manual preoperative planning for primary shoulder arthroplasty requires experience and may be time consuming^12^; and radiographic evaluation often fails to produce reliable results.^13^ For revision shoulder arthroplasty, planning is even more challenging due to difficulties in assessing remaining glenoid and humeral bone stock as a result of the metal artifact.^14^^,^^23^ Based on our findings, this new software feature provides valuable support to surgeons in addressing these challenges.

A limitation to our study is that this survey research was focused on surgeon satisfaction and perceived benefits of the new software feature. No clinical outcome data were collected as the survey was limited to the preoperative planning phase and an intraoperative evaluation. However, all respondents to our survey are fellowship-trained and well-experienced orthopedic surgeons. Therefore, our findings may provide a valid representation of the current clinical opinion in shoulder arthroplasty and the current clinical practice of revision surgery. On the other hand, this survey research was addressed to a limited number of surgeons only, and all respondents had previously used Blueprint in their clinical practice; the generalizability of survey results may thus be severely limited. Clinical investigations should be conducted to investigate with greater scientific rigor how the use of the new software feature for preoperative planning of revision and complex primary shoulder arthroplasty cases affects patient’s clinical outcomes or impacts the cost.

## Conclusion

The use of Blueprint for preoperative 3D planning of revision shoulder arthroplasty cases rendered positive feedback from experienced orthopedic surgeons when surveyed under real-world conditions. Perceived benefits that relate to the preoperative planning phase include increased efficiency and improved ease of creating an appropriate plan, while also increasing the surgeon’s confidence to execute the surgical procedure according to the plan. In the intraoperative evaluation, use of the new Blueprint feature was perceived especially beneficial in terms of an appropriate implant selection and accurate implant placement. Surgeon satisfaction and overall ease of use ratings were broadly favorable, both in the preoperative planning phase as well as in the intraoperative evaluation. Further research should be conducted to investigate whether these perceived benefits can lead to improved patient’s clinical outcomes or impacts the cost associated with revision shoulder arthroplasty.

## Acknowledgment

The authors thank Arthur de Gast, MD, PhD, and Trystan Louboutin for providing support with the analysis of imaging files. The authors also thank Jolanda Boutesteijn and Maud Reynier for providing assistance and helpful comments on earlier drafts of the manuscript.

## Disclaimers:

Funding: The conduct of the research project and the article publishing charges were financed by 10.13039/100008894Stryker.

Conflicts of interest: Matthias Regling is an employee of Stryker Trauma GmbH and a shareholder of Stryker Corporation. Brendan M. Patterson, MD, MPH is a paid consultant for Styker Orthopedics and receives research funding from Stryker Orthopedics.
